# Characteristics and risk factors of urinary tract infection in patients with HBV-related acute-on-chronic liver failure: A retrospective study

**DOI:** 10.1097/MD.0000000000029913

**Published:** 2022-07-15

**Authors:** Qian Zhang, Baoxian Shi, Liang Wu

**Affiliations:** a Division of Nephrology, Department of Internal Medicine, Tongji Hospital, Tongji Medical College, Huazhong University of Science and Technology, Wuhan, Hubei, People’s Republic of China; b Department of Chemistry and Environmental Engineering, Wuhan Polytechnic University, Wuhan, Hubei, People’s Republic of China; c Department and Institute of Infectious Disease, Tongji Hospital, Tongji Medical College, Huazhong University of Science and Technology, Wuhan, Hubei, People’s Republic of China.

**Keywords:** acute-on-chronic liver failure, hepatitis B virus, prevalence, risk factors, urinary tract infection

## Abstract

Acute-on-chronic liver failure (ACLF) is a syndrome characterized by acute decompensation, organ failures, and high short-term mortality. Hepatitis B virus (HBV) is the main cause of liver failure in China. Urinary tract infection (UTI) is one of the common bacterial infections in patients with HBV-ACLF. However, few studies concerning the risk factors and epidemiology have been published.

A retrospective analysis of 539 patients with HBV-ACLF was performed. The prevalence, bacterial profile, and antibiotic susceptibility pattern were investigated and associated risk factors of UTI in patients with HBV-ACLF were evaluated with a logistic regression model.

The overall prevalence of UTI among the study participants was 26.53% (143/539), and 64.34% (92/143) of them were asymptomatic. One hundred thirty-five strains of bacteria, including 74.07% (100/135) gram-negative bacteria and 53.33% (72/135) multidrug-resistant organisms, were cultivated from 143 patients with HBV-ACLF. *Escherichia coli* 46.67% (63/135) and *Klebsiella pneumoniae* 13.33% (18/135) were the most common bacteria. The antibiotic susceptibility test pattern showed that 92.93%, 81.63%, and 81.63% of the gram-negative isolates were sensitive to imipenem, tigecycline, and piperacillin/tazobactam, respectively. Meanwhile, all the gram-positive isolates were sensitive to linezolid, teicoplanin, and vancomycin. Compared with non-UTI group, the patients with UTI had higher serum creatinine, lower educational status, total bilirubin, direct bilirubin, and albumin. Finally, educational status and albumin were independent risk factors in the prevalence of UTI in patients with HBV-ACLF.

UTI is one of the common bacterial infections seen in patients with HBV-ACLF. Gram-negative bacteria account for the majority of cultured bacteria, and multidrug-resistant bacteria are common. UTI is determined by a diverse set of complex factors, which lower educational status and hypoalbuminemia predict the more prevalence of UTI.

## 1. Introduction

Liver failure is a serious acute or chronic liver insufficiency induced by a variety of causes, which has high mortality. However, there is high diversity in the definition, etiology, classification, diagnosis, and treatment of liver failure in different countries.^[1]^ Liver failure is classified into acute liver failure (ALF), subacute liver failure (SALF), acute-on-chronic liver failure (ACLF), and chronic liver failure. Acute hepatic insults of infectious etiology included reactivation of hepatitis B virus (HBV) as the leading cause of ACLF in the Asian region.^[2]^ Among the noninfectious etiologies, alcoholic hepatitis is the major cause of acute deterioration in stable known or unknown chronic liver diseases, more often in western countries.^[2,3]^ Liver failure can develop further deterioration of decompensated end-stage liver disease (ESLD) without appropriate interventions. For patients with ESLD, liver transplantation is the only effective treatment, which carries a huge burden for families.^[4]^ However, patients with ALF, SALF, and ACLF may recover with early diagnosis, etiological determination, and therapy.

ACLF is a clinical syndrome of acute liver decompensation resulting from a precipitating event (PE) in patients with previously compensated liver disease.^[5]^ ACLF is very frequent, affecting 30% to 40% of hospitalized patients, and is associated with high short-term mortality rate (30% at 28 days).^[6]^ In China, HBV-related ACLF is a leading cause of liver failure because of the high incidence of chronic HBV infection.^[4,7]^ It has been reported that PEs of bacterial infection, HBV reactivation, active alcoholism, and superimposed hepatitis virus infection were risk factors for ACLF.^[8]^ Some studies also revealed that bacterial infection was present in about one-third of ACLF patients at presentation to a tertiary referral hospital, and this further increased by the first week.^[2,9]^ Common bacterial infections include spontaneous bacterial peritonitis (SBP), urinary tract infection (UTI), community-acquired pneumonia, dermatologic infection, and bacteremia.^[10]^ Prompt detection of infections and use of antimicrobial agents for treatment can help improve survival in these patients. However, surveys on bacterial infections primarily focus on SBP. Sufficient data are lacking on ACLF patients with UTI, which is considered one of the most common complications. Moreover, antibiotic resistance of urinary tract pathogens has been increasing worldwide, especially to the commonly used antimicrobials.^[11]^ It is worth discussing how to treat ACLF patients with UTI accurately. Therefore, based on a retrospective analysis of 539 patients with ACLF, this study was designed to investigate the uropathogen profile, antibiotic susceptibility pattern, and associated risk factors of UTI in patients with HBV-related ACLF.

## 2. Materials and Methods

### 2.1. Ethics approval and consent to participate

This research project was performed in accordance with the principles of the Helsinki Declaration. The Ethics Research Committee at Tongji Hospital approved the study (TJ-IRB20190601). The information of all patients was anonymized and de-identified prior to analysis.

### 2.2. Participants and design

The retrospective study was performed in the department and institute of infectious disease of Tongji Hospital in China. Data from 874 patients with HBV-ACLF were reviewed from Oct 2015 to May 2018. The inclusion criteria were as follows: the age of the patients was >18 years old; all patients were met the consensus recommendations of the Asian Pacific association for the study of the liver (APASL): ACLF is an acute hepatic insult manifesting as jaundice (serum bilirubin ≥5 mg/dL (85 mmol/L) and coagulopathy (INR ≥1.5 or prothrombin activity <40%) complicated within 4 weeks by clinical ascites and/or encephalopathy in a patient with previously diagnosed or undiagnosed chronic liver disease/cirrhosis, and is associated with high 28-day mortality.^[2]^ All the patients have received antivirus medication for HBV, including telbivudine, entecavir, or tenofovir. Patients were excluded as follows: coinfection with other viruses (hepatitis A, C, D, or E) or human immunodeficiency virus (HIV) and syphilis; hepatic disease in schistosomiasis, alcoholic hepatic disease, drug-induced hepatitis, fatty liver disease, autoimmune liver disease, Wilson disease, and hepatocellular carcinoma (HCC); SLE, pregnant patients, and postoperative patients; the instrumentation of patients with urinary catheters; patients had no record of urine routine. Information on prognosis was verified through medical records.

### 2.3. Clinical and laboratory data collection

Information regarding the age, gender, body mass index, education level, course of hepatitis, medication used (including diuretics and antibiotics), copies of HBV DNA, severe complications, and laboratory tests were recorded. The laboratory parameters included prothrombin activity, alanine aminotransferase, aspartate transaminase, total bilirubin (TBIL), direct bilirubin (DBIL), albumin, globulin, total cholesterol, triglyceride, serum creatinine (SCr), blood urea nitrogen, serum uric acid, HCO_3_^–^, white blood cell (WBC) count, hemoglobin, and platelet. Urine specimens were collected from patients with HBV-ACLF when admitted to inpatient department at first day; UTI was defined as follows: a combination of urine white blood count >11 cells/μL in males and 35 cells/μL in females with positive culture or uncountable leucocytes per field if negative cultures.^[12]^ Multidrug-resistant (MDR) bacteria was defined as acquired nonsusceptibility to at least 1 agent in 3 or more antimicrobial categories. Extensively drug-resistance (XDR) bacteria was defined as nonsusceptibility to at least 1 agent in all but 2 or fewer antimicrobial categories.^[13]^ In order to facilitate statistics, the parameters were defined as follows: education level, low = illiteracy and primary school, middle = junior school and senior school, high = more than college degree; HBV DNA, negative = ≤10^3^ copies for HBsAg-negative patients, and ≤10^4^ copies for HBsAg-positive patients; low level = 10^3^ to 10^7^ copies for HBsAg-negative patients and 10^4^ to 10^7^ copies for HBsAg-positive patients; and high level = ≥10^7^ copies for both HBsAg-negative and HBsAg-positive patients.

### 2.4. Urine specimen collection, bacteriological investigation, and antibiotic susceptibility testing

Urine specimens were obtained by the clean catch-voided midstream technique for laboratory examination at least 3 times when patients with HBV-ACLF were admitted to inpatient department at first day. All urine samples were inoculated onto cysteine lactose electrolyte-deficient medium and mannitol salt agar. After incubation at 37°C for 18- to 24-hour colonies were counted to check for significant growth. Colony counts yielding bacterial growth of 10^5^ colony-forming unit/mL were regarded as significant for bacteriuria. Colonies from cysteine lactose electrolyte-deficient medium were subcultured into MacConkey agar and blood agar plates (BAP) and incubated at 37°C for 18 to 24 hours. Bacteria were identified using colony characteristics, gram reaction of the organisms, and biochemical tests following standard procedure. Antibiotic susceptibility testing was performed on all significant isolates according to the criteria of Clinical and Laboratory Standards Institute. Agar disc diffusion method was used to determine susceptibility of the isolates. Individual colonies were suspended in 5 mL normal saline to 0.5 McFarland standard in order to standardize the inoculum size and the suspensions were evenly distributed over the entire surface of Muller Hinton agar. The antibiotic discs were placed on the inoculated plates and incubated at 37°C for 18 to 24 hours. Diameter of the zone of inhibition around the disc was measured using a digital metal caliper and isolates were classified as sensitive, intermediate, and resistant according to Clinical and Laboratory Standards Institute. All procedures were conducted in the Laboratory Department of Tongji Hospital, Huazhong University of Science and Technology.

### 2.5. Data analysis

All statistical analyses were performed using the SPSS 20.0 software. The categorical data were reported as numbers and percentages. Continuous variables were summarized as means ± standard deviation. Differences among groups were compared with Student *t* test for the normally distributed variables, the χ^2^ test for the categorical data and Mann–Whitney *U* test for the nonnormally distributed variables. Logistic regression analysis was used for modeling the relationship between UTI and clinical characteristics. The *P* values reported were 2-sided and taken to be significant at <.05.

## 3. Results

### 3.1. Characteristics of all patients with HBV-ACLF

**Figure 1.**
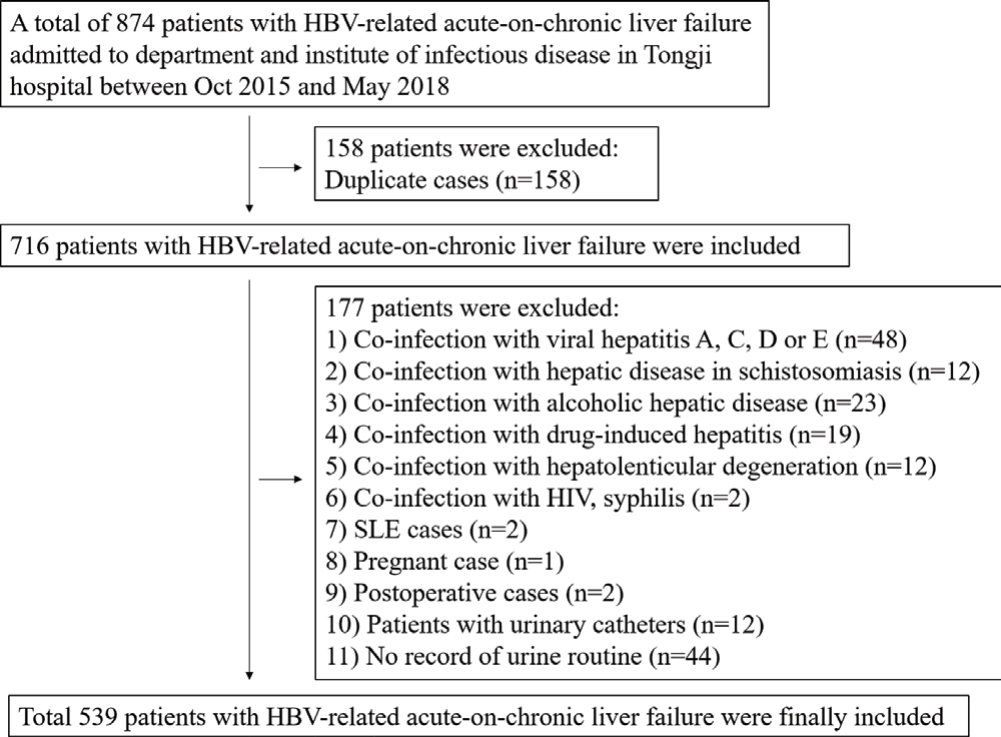
Participant flow for the study.

Between October 2015 and May 2018, 874 adult patients admitted to the department and institute of infectious disease were screened for eligibility according to the inclusion and exclusion criteria. A total of 539 patients with HBV-ACLF were finally included (Fig. 1).

**Figure 2.**
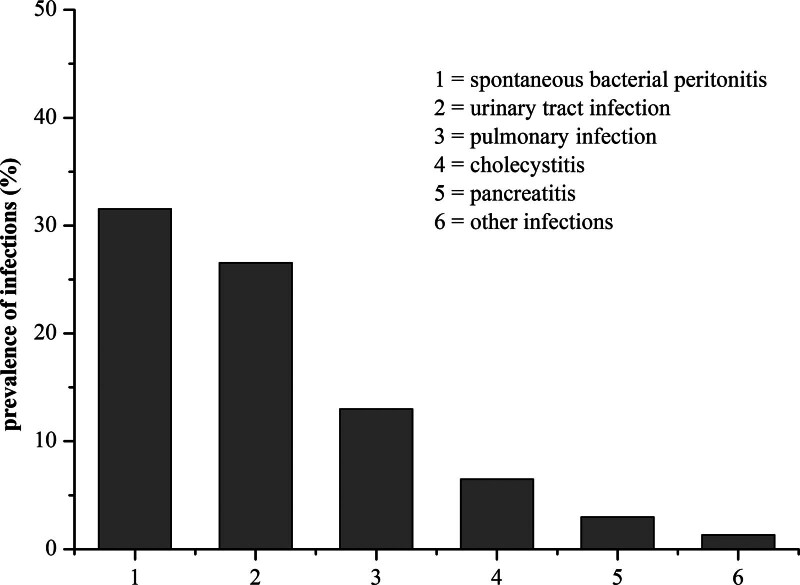
The prevalence of types of infections in patients with HBV-ACLF. ACLF = acute-on-chronic liver failure, HBV = hepatitis B virus.

Patients with HBV-ACLF were often at higher risk of getting infected, for example, upper respiratory tract infection, UTI, and digestive tract infections. In our study, the prevalence of infections in different sites was investigated among patients with severe HBV. SBP was the most common (31.54%; 170/539), and then followed by UTI (26.53%; 143/539), pulmonary infection (12.99%; 70/539), cholecystitis (6.49%; 35/539), pancreatitis (2.97%; 16/539), and other infections (1.3%; 7/539; Fig. 2).

Of the 539 patients, 468 were males and 71 were females. The mean age was 44.88 ± 11.50 years. The baseline demographic, clinical, and laboratory characteristics of the patients with HBV-ACLF are summarized in Table 1.

**Table 1:** Baseline demographic, clinical, and laboratory characteristics of the study population.

	All	Urinary tract infection group	Nonurinary tract infection group	*P* value
No. of patients	539	143	396	
Age (yr)	44.88 ± 11.50	45.82 ± 11.81	44.55 ± 11.39	.257
Gender				
Male, n (%)	468 (86.83)	119 (83.22)	349 (88.13)	Reference
Female, n (%)	71 (13.17)	24 (16.78)	47 (11.87)	.150
BMI (kg/m^2^)	23.45 ± 3.99	23.75 ± 4.16	23.34 ± 3.93	.283
Education level, n (%)				
Low level	94 (17.44)	39 (27.27)	55 (13.89)	Reference
Middle level	342 (63.45)	92 (64.34)	250 (63.13)	.008
High level	103 (19.11)	12 (8.39)	91 (22.98)	<.001
Course of hepatitis (yr)	10.41 ± 9.52	10.17 ± 8.99	10.50 ± 9.71	.721
Quantity of diuretics, n (%)				
0	148 (27.46)	34 (23.78)	114 (28.79)	Reference
1	17 (3.15)	5 (3.50)	12 (3.03)	.553
2	314 (58.26)	87 (60.84)	227 (57.32)	.308
≥ 3	60 (11.13)	17 (11.89)	43 (10.86)	.477
Frequency of diuretics, n (%)				
0	148 (27.46)	34 (23.78)	114 (28.79)	Reference
Once a day	44 (8.16)	12 (8.39)	32 (8.08)	.552
Twice a day	54 (10.02)	15 (1.79)	39 (9.85)	.578
Three times a day	293 (54.36)	82 (57.34)	211 (53.28)	.303
Other infections, n (%)				
0	295 (54.73)	77 (53.85)	218 (55.05)	Reference
1	179 (33.21)	46 (32.17)	133 (33.59)	.506
2	56 (10.39)	17 (11.89)	39 (9.85)	.306
≥ 3	9 (1.67)	3 (2.10)	6 (1.52)	.437
Quantity of antibiotic, n (%)				
0	23 (4.27)	8 (5.59)	15 (3.79)	Reference
1	150 (27.83)	50 (34.97)	100 (25.25)	.532
2	135 (25.05)	31 (21.68)	104 (26.26)	.169
3	97 (18.00)	20 (13.99)	77 (19.44)	.123
≥ 4	134 (24.86)	34 (23.78)	100 (25.25)	.242
Therapeutic time of antibiotics, d, n (%)				
0	23 (4.27)	8 (5.59)	15 (3.79)	Reference
1–10	100 (18.55)	33 (23.08%)	67 (16.92)	.526
11–20	123 (22.82)	29 (20.28)	94 (23.74)	.189
21–30	97 (18.00)	22 (15.38)	75 (18.94)	173
≥31	196 (36.36)	51 (35.66)	145 (36.62%)	0.253
Hepatic encephalopathy, n (%)				
Negative	416 (77.18)	116 (81.12)	300 (75.76)	Reference
Positive	123 (22.82)	27 (18.88)	96 (24.24)	.203
Hepatorenal syndrome, n (%)				
Negative	460 (85.34)	120 (83.92)	340 (85.86)	Reference
Positive	79 (14.66)	23 (16.08)	56 (14.14)	.582
Cirrhosis, n (%)				
Negative	347 (64.38)	88 (61.54)	259 (65.40)	Reference
Positive	192 (35.62)	55 (38.46)	137 (34.60)	.417
Long-time bed, n (%)				
Negative	282 (52.32)	75 (52.45)	207 (52.27)	Reference
Positive	257 (47.68)	68 (47.55)	189 (47.73)	.525
HbeAg, n (%)				
Negative	361 (66.98)	92 (64.34)	269 (67.93)	Reference
Positive	178 (33.02)	51 (35.66)	127 (32.07)	.248
HBV DNA, n (%)				
Negative	125 (23.19)	30 (20.98)	95 (23.99)	Reference
Low level copies	309 (57.33)	89 (62.24)	220 (55.56)	.343
High level copies	105 (19.48)	24 (16.78)	81 (20.45)	.877
Laboratory tests				
PTA (%)	31.54 ± 6.62	31.15 ± 7.06	31.68 ± 6.45	.646
ALT (U/L)	491.50 ± 584.79	438.26 ± 468.68	510.73 ± 620.83	.149
AST (U/L)	363.53 ± 408.02	341.06 ± 347.40	371.64 ± 427.93	.443
TBIL (μmol/L)	300.59 ± 137.81	282.09 ± 137.02	307.28 ± 137.66	.061
DBIL (μmol/L)	214.21 ± 100.13	201.46 ± 98.64	218.81 ± 100.39	.076
ALB (g/L)	30.90 ± 4.98	27.89 ± 3.50	31.99 ± 4.99	<.001
Globulin (g/L)	28.42 ± 7.58	28.46 ± 6.26	28.40 ± 8.01	.935
TC (mmol/L)	2.31 ± 0.89	2.29 ± 0.97	2.32 ± 0.86	.772
TG (mmol/L)	1.16 ± 0.69	1.14 ± 0.45	1.17 ± 0.76	.652
SCr (μmol/L)	77.72 ± 50.27	84.19 ± 61.98	75.38 ± 45.17	.072
BUN (mmol/L)	5.38 ± 4.94	5.69 ± 4.89	5.27 ± 4.96	.391
UA (μmol/L)	186.58 ± 126.65	194.52 ± 120.23	183.70 ± 128.93	.382
HCO_3_– (mmol/L)	24.00 ± 3.64	23.71 ± 3.53	24.10 ± 3.67	.275
WBC (×10^9^/L)	6.92 ± 3.58	6.99 ± 4.40	6.90 ± 3.24	.782
Hb (g/L)	123.31 ± 22.55	122.36 ± 23.48	123.65 ± 22.22	.556
PLT (×10^9^/L)	110.95 ± 58.39	113.68 ± 62.01	109.96 ± 57.08	.513

Results are reported as means ± standard deviation.

ALB = albumin, ALT = alanine aminotransferase, AST = aspartate transaminase, BMI = body mass index, BUN = blood urea nitrogen, DBIL = direct bilirubin, Hb = hemoglobin, HbeAg = hepatitis B e antigen, HBV = hepatitis B virus, PLT = platelet, PTA = prothrombin activity, SCr = serum creatinine, TBIL = total bilirubin, TC = total cholesterol, TG = triglyceride, UA = uric acid, WBC = white blood cell.

### 3.2. Characteristics of uropathogens in patients with UTI

The overall prevalence of UTI among the study participants was 26.53% (143/539). Furthermore, 64.34% (92/143) of them were asymptomatic and 35.66% (51/143) were symptomatic. A total of 135 isolates were detected in urine samples of the patients with UTI. Culture results revealed that the proportion of gram-negative bacteria, gram-positive bacteria, and fungi were respectively 74.07% (100/135), 17.04% (23/135), and 8.89% (12/135). *Escherichia coli* 46.67% (63/135) was the most common causative organism of the infection. *Klebsiella pneumoniae* 13.33% (18/135), *Enterococcus faecalis* 8.89% (12/135), *Enterococcus faecium* 5.19% (7/135), *Acinetobacter baumanii* 3.70% (5/135), and *Pseudomonas aeruginosa* 2.96% (4/135) were the moderately identified bacterial species (Tables 2 and 3). A total of 12 fungi were detected, including *Candida tropicalis* 5.19% (7/135), *Candida albicans* 2.22% (3/135), and *Candida glabrata* 1.48% (2/135). Five of the isolates were found in mixed growth, including *E coli + A baumanii*, *E coli + P aeruginosa*, *E coli + C tropicalis*, *E coli + C albicans*, and *K pneumoniae* + *C tropicalis* (data not shown).

**Table 2:** Prevalence of isolated gram-negative uropathogens and antibiotic susceptibility pattern of bacterial isolates in patients with urinary tract infection.

Bacterial species	Total n (%)	S/ I/ R	Antibiotic discs tested									
			CAZ n (%)	CTX n (%)	F n (%)	IPM n (%)	LEV n (%)	PRL n (%)	SCF n (%)	SXT n (%)	TGC n (%)	TZP n (%)
*Escherichia coli*	63 (49.32)	S	37 (58.73)	19 (30.16)	50 (79.37)	63 (100)	25 (39.68)	10 (15.87)	46 (73.01)	26 (41.27)	58 (92.06)	56 (88.89)
		I	5 (7.94)	1 (1.59)	6 (9.52)	–	1 (1.59)	4 (6.35)	12 (19.05)	–	4 (6.35)	6 (9.52)
		R	21 (33.33)	43 (68.25)	7 (11.11)	–	37 (58.73)	49 (77.78)	5 (7.94)	37 (58.73)	1 (1.59)	1 (1.59)
*Klebsiella pneumoniae*	18 (12.84)	S	12 (66.67)	9 (50)	8 (44.44)	17 (94.44)	13 (72.22)	7 (38.89)	9 (50)	9 (50)	10 (55.56)	13 (72.22)
		I	2 (11.11)	1 (5.56)	5 (27.78)	–	–	1 (5.56)	7 (38.89)	–	5 (27.78)	1 (5.56)
		R	4 (22.22)	8 (44.44)	5 (27.78)	1 (5.56)	5 (27.78)	10 (55.56)	2 (11.11)	9 (50)	3 (16.67)	4 (22.22)
–*Acinetobacter baumanii*	5 (4.05)	S	3 (60)	2 (40)	–	3 (60)	3 (60)	2 (40)	2 (40)	2 (40)	3 (60)	2 (40)
		I	–	1 (20)	–	–	–	1 (20)	1 (20)	–	1 (20)	–
		R	2 (40)	2 (40)	–	2 (40)	2 (40)	2 (40)	2 (40)	3 (60)	1 (20)	3 (60)
*Pseudomonas aeruginosa*	4 (3.38)	S	3 (75)	3 (75)	–	3 (75)	3 (75)	3 (75)	2 (50)	1 (25)	2 (50)	4 (100)
		I	–	–	–	–	–	1 (25)	1 (25)	–	1 (25)	–
		R	1 (25)	1 (25)	–	1 (25)	1 (25)	–	1 (25)	3 (75)	1 (25)	–
*Citrobacter freundii*	2 (1.35)	S	1 (50)	1 (50)	2 (100)	2 (100)	2 (100)	1 (50)	2 (100)	2 (100)	2 (100)	1 (50)
		I	–	–	–	–	–	–	–	–	–	1 (50)
		R	1 (50)	1 (50)	–	–	–	1 (50)	–	–	–	–
*Chryseobacterium indologenes*	1 (0.68)	S	–	–	–	–	–	–	–	1 (100)	1 (100)	–
		I	–	–	–	–	–	–	–	–	–	–
		R	1 (100)	1 (100)	–	1 (100)	1 (100)	1 (100)	1 (100)	–	–	1 (100)
*Elizabethkingia meningoseptica*	1 (0.68)	S	–	–	–	–	–	–	–	1 (100)	1 (100)	1 (100)
		I	–	–	–	–	–	1 (100)	–	–	–	–
		R	1 (100)	1 (100)	–	1 (100)	1 (100)	–	1 (100)	–	–	–
*Proteus mirabillis*	1 (0.68)	S	1 (100)	–	–	1 (100)	–	1 (100)	1 (100)	–	1 (100)	1 (100)
		I	–	1 (100)	–	–	1 (100)	–	–	–	–	–
		R	–	–	1 (100)	–	–	–	–	1 (100)	–	–
*Stenotrophomonas maltophilia*	1 (0.68)	S	–	–	–	–	1 (100)	–	–	1 (100)	1 (100)	-
		I	–	–	–	–	–	–	–	–	–	–
		R	–	–	–	–	–	–	–	–	–	–
*Burkholderia cepacia*	1 (0.68)	S	1 (100)	–	–	1 (100)	–	–	–	1 (100)	1 (100)	–
		I	–	–	–	–	–	–	–	–	–	–
		R	–	–	–	–	–	–	–	–	–	–
*Enterobacter cloacae*	1 (0.68)	S	1 (100)	–	–	1 (100)	–	–	–	–	–	–
		I	–	1 (100)	–	–	–	–	–	–	–	1 (100)
		R	–	–	1 (100)	–	1 (100)	1 (100)	1 (100)	1 (100)	1 (100)	–
*Pseudomonas fluorescens*	1 (0.68)	S	1 (100)	1 (100)	–	–	–	–	1 (100)	1 (100)	–	1 (100)
		I	–	–	–	–	-	1 (100)	–	–	–	–
		R	–	–	–	1 (100)	1 (100)	–	–	–	–	–
*Serratia marcescens*	1 (0.68)	S	1 (100)	1 (100)	–	1 (100)	1 (100)	1 (100)	1 (100)	1 (100)	–	1 (100)
		I	–	–	–	–	–	–	–	–	–	–
		R	–	–	1 (100)	–	–	–	–	–	–	–
Total	100 (76.35)	S	61 (61.62)	36 (36.73)	60 (69.77)	92 (92.93)	48 (48.48)	25 (25.51)	64 (65.31)	46 (46)	80 (81.63)	80 (81.63)
		I	7 (7.07)	5 (5.1)	11 (12.79)	–	2 (2.02)	9 (9.18)	21 (21.43)	–	11 (11.22)	9 (9.18)
		R	31 (31.31)	57 (58.16)	15 (17.44)	7 (7.07)	49 (49.49)	64 (65.31)	13 (13.27)	54 (54)	7 (7.14)	9 (9.18)

CAZ = ceftazidime, CTX = cefotaxime, *E coli* = *Escherichia coli*, F= nitrofurantoin, I = intermediate, IPM = imipenem, *K pneumonia* = *Klebsiella pneumonia*, LEV = levofloxacin, PRL = PIperacillin, R = resistant, S = sensitive, SCF = cefoperazone/sulbactam, SXT = sulfamethoxazole and trimethoprim, TGC = tigecycline, TZP = piperacillin/tazobactam.

**Table 3:** Prevalence of isolated gram-positive uropathogens and antibiotic susceptibility pattern of bacterial isolates in patients with urinary tract infection

Bacterial species	Total n (%)	S/ I/ R	Antibiotic discs tested							
			AMP n (%)	F n (%)	LEV n (%)	LZD n (%)	PG n (%)	TEC n (%)	TGC n (%)	VA n (%)
*Enterococcus faecalis*	12 (8.11)	S	10 (83.33)	8 (66.67)	9 (75)	12 (100)	10 (83.33)	12 (100)	11 (91.67)	12 (100)
		I	–	2 (16.67)	–	–	–	–	1 (8.33)	–
		R	2 (16.67)	2 (16.67)	3 (25)	–	2	–	–	–
*Enterococcus faecium*	7 (4.73)	S	–	2 (28.57)	1 (14.29)	7 (100)	–	7 (100)	5 (71.43)	7 (100)
		I	–	1 (14.29)	–	–	–	–	1 (14.29)	–
		R	7 (100)	4 (57.14)	6 (85.71)	–	7 (100)	–	1 (14.29)	–
*Staphylococcus aureus*	2 (1.35)	S	–	–	1 (50)	2 (100)	–	2 (100)	2 (100)	2 (100)
		I	–	–	–	–	–	–	–	–
		R	2 (100)	2 (100)	1 (50)	–	2 (100)	–	–	–
*Streptococcus viridans*	1 (0.68)	S	1 (100)	–	1 (100)	1 (100)	1 (100)	1 (100)	1 (100)	1 (100)
		I	–	1 (100)	–	–	–	–	–	–
		R	–	–	–	–	–	–	–	–
*Streptococcus agalactiae*	1 (0.68)	S	1 (100)	-	1 (100)	1 (100)	–	1 (100)	1 (100)	1 (100)
		I	–	1 (100)	–	–	–	–	–	–
		R	–	–	–	–	1 (100)	–	–	–
Total	23 (17.04)	S	12 (52.17)	10 (43.48)	13 (56.52)	23 (100)	11 (47.83)	23 (100)	20 (86.96)	23 (100)
		I	–	5 (21.74)	–	–	–	–	2 (8.7)	–
		R	11 (47.83)	8 (34.78)	10 (43.48)	–	12 (52.17)	–	1 (4.35)	–

AMP = ampicillin, F = nitrofurantoin, I = intermediate, LEV = levofloxacin, LZD = linezolid, PG = penicillin G, R = resistant, S = sensitive, TEC = teicoplanin, TGC = tigecycline, VA = vancomycin.

### 3.3. Antibiotic susceptibility test pattern

The antibiotic susceptibility test pattern showed that 92.93%, 81.63%, and 81.63% of the gram-negative isolates were sensitive to imipenem, tigecycline, and piperacillin/tazobactam, respectively. 65.31%, 58.16%, and 49.49% of the isolates were resistant to piperacillin, cefotaxime, and levofloxacin, respectively. All *E coli* isolates had shown sensitivity to imipenem. Conversely, *K pneumoniae* was the isolate to show more sensitivity to piperacillin/tazobactam, levofloxacin, and ceftazidime than tigecycline. Nearly half of *A. baumanii* isolates were resistant to the tested antibiotics. The results also showed that all the gram-positive isolates were sensitive to linezolid, teicoplanin, and vancomycin. 52.17%, 47.83%, and 43.48% of the isolates were resistant to penicillin G, ampicillin, and levofloxacin, respectively. High level of resistance was observed in *E faecium* and *S aureus* isolates (Tables 2 and 3). Moreover, antibiotic susceptibility test pattern reveled that 53.33% (72/135) MDR bacteria and 5.19% (7/135) XDR were found in all uropathogens. All fungi were sensitive to fluconazole (data not shown).

### 3.4. The characteristics of anti-infective therapy in patients with UTI

**Figure 3.**
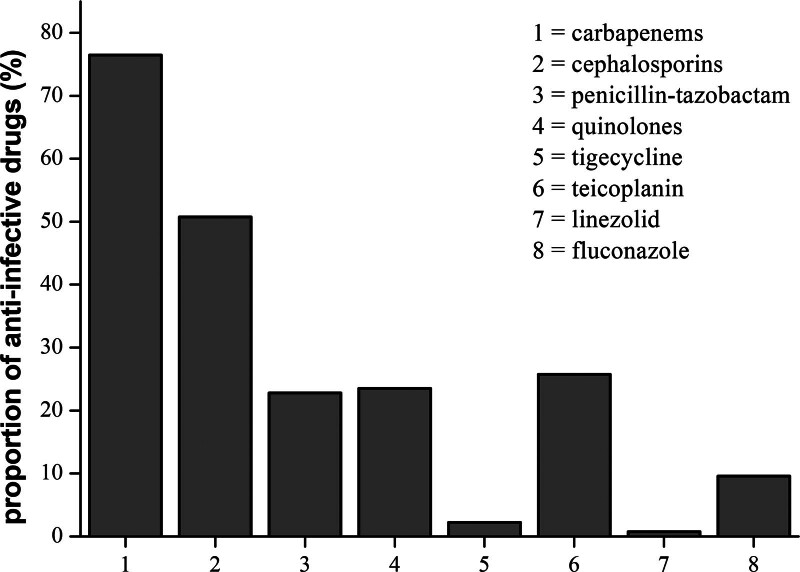
The types and proportions of anti-infective therapy in patients with UTI. UTI = urinary tract infection.

Among the 143 patients with UTI, 95.1% (136/143) were received anti-infective therapy. 34.27% (49/143) patients received single antibiotics, while the others were involved with combined drugs. In the single-antibiotic therapy, 65.31% (32/49) were carbapenems, 30.61% (15/49) were third-generation cephalosporins or piperacillin-tazobactam, and the remaining 4.08% (2/49) were quinolones. Among all the patients with UTI who received anti-infective therapy, 76.47% (104/136) were carbapenems, 50.74% (69/136) were cephalosporins, 22.79% (31/136) were penicillin-tazobactam, 23.53% (32/136) were quinolones, 2.21% (3/136) were tigecycline, 25.74% (35/136) were teicoplanin, 0.74% (1/136) were linezolid, and 9.56% (13/136) were fluconazole. The results are shown in Figure 3.

### 3.5. Determinants of UTI in patients with HBV-ACLF

Compared with non-UTI group, the patients with UTI had higher SCr, lower educational status, TBIL, DBIL, and albumin. With UTI as a dependent variable and the above factors as covariates, only 2 variables, educational status and albumin, were independent risk factors in the prevalence of UTI in patients with HBV-ACLF. According to logistic regression analyses, middle and high educational status were associated with a lower risk of UTI than lower educational status (odds ratios for middle and high educational status were 0.744 and 0.250, respectively) after adjustment for gender, age, and body mass index. The higher quartiles of albumin (quartiles 2–4) were associated with a lower risk of UTI than quartile 1 (odds ratios for quartiles 2–4 were 0.560, 0.160, and 0.030, respectively). The multivariate determinants for UTI among individuals with HBV-ACLF are shown in Table 4.

**Table 4:** Independent risk factors in LR for UTI among individuals with ACLF.

Determinants	Quartile	Model 1	Model 2
		OR (95% CI)	OR (95% CI)
Education level			
Low level		Reference	Reference
Middle level		0.772 (0.425–1.400)	0.744 (0.405–1.366)
High level		0.257 (0.109–0.610)^**^	0.250 (0.104–0.602)^**^
ALB	1	Reference	Reference
	2	0.612 (0.359–1.043)^*^	0.560 (0.324–0.969)^*^
	3	0.169 (0.087–0.326)^**^	0.160 (0.081–0.316)^**^
	4	0.040 (0.014–0.116)^**^	0.030 (0.009–0.099)^**^

The cutoffs for the quartiles of ALB were 28.2 g/L, 31.1 g/L and 34.4 g/L. Model 1 was calculated using logistic regression and model 2 was adjusted for gender, age and BMI.

ACLF = acute-on-chronic liver failure, ALB = albumin, BMI = body mass index, CI = confidence interval, LR= logistic regression, OR = odds ratio, UTI = urinary tract infection.

* *P* < .05,

** *P* < .01.

### 3.6. Prognosis in patients with HBV-ACLF

Among the 143 patients with UTI, 32.87% (47/143) were discharged after improvement, 44.06% (63/143) were discharged automatically for financial, medical insurance or personal reasons, 15.38% (22/143) developed pyuria, 0.7% (1/143) were received liver transplantation, and 6.99% (10/143) died. Meanwhile, among the 396 patients with UTI, 44.95% (178/396) were discharged after improvement, 43.94% (174/396) were discharged automatically for financial, medical insurance or personal reasons, 3.03% (12/396) were in coma, 2.02% (8/396) were received liver transplantation, and 6.06% (24/396) died. The results are shown in Table 5.

**Table 5:** Outcomes in patients with HBV-ACLF in UTI group and non-UTI group.

Outcomes	UTI group (N = 143)	Non-UTI group (N = 396)	*P* value
Better discharge, n (%)	47 (32.87)	178 (44.95)	.012
Automatic discharge,^*^ n (%)	63 (44.06)	174 (43.94)	.981
Pyuria, n (%)	22 (15.38)	–	
Liver transplantation, n (%)	1 (0.7)	8 (2.02)	.499
Coma, n (%)	–	12 (3.03)	
Death, n (%)	10 (6.99)	24 (6.06)	.694

ACLF = acute-on-chronic liver failure, HBV = hepatitis B virus, UTI = urinary tract infection.

* Including patients who were transferred to other hospitals, went home, and gave up treatment.

## 4. Discussion

HBV infection is a global public health issue, because persistent viremia is associated with increased morbidity and mortality.^[14]^ However, the prevalence of HBV infection varies widely in different regions. The World Health Organization reported that >2 billion people currently live with serologic evidence of a past or present HBV infection. About 250 million of these people are chronically infected, among which up to 40% may develop liver complications, including cirrhosis, liver decompensation, and HCC.^[15–17]^ In patients with cirrhosis and HCC in our country, the proportion of HBV infection was 60% and 80%, respectively.^[18]^ In China, with the development of HBV-related liver failure, the epidemiology and classification differ a variety of changes in the past 10 years. The incidence of ALF and SALF has gradually decreased due to expanded immunization and effective antiviral therapy; meanwhile, ACLF and CLF have gradually increased because of types of PEs. As the research reported, ACLF was a leading type of liver failure, accounting for 3429 cases in 3916 patients (87.6%). HBV infection accounted for 87.3% of ACLF cases owing to a high incidence of chronic HBV infection.^[7]^

The research has reported that bacterial infection was a main precipitating factor in patients with ACLF.^[8]^ Meanwhile, bacterial infections were severe and associated with intense systemic inflammation, poor clinical course and high mortality in patients with ACLF.^[19]^ In our investigation, 321 cases (59.55%) in total 539 patients showed evidence of infections, including SBP, UTI, pulmonary infection, cholecystitis, pancreatitis, and other infections. Among them, SBP (31.54%), UTI (26.53%), and pulmonary infection (12.99%) were some of the common bacterial infections seen in patients with HBV-ACLF. Unlike the UTI in the general population, most patients with HBV-ACLF were asymptomatic, including dysuria, urinary frequency, and urgency, which can give rise to a diagnostic dilemma. In fact, the identical problem also existed in other ESLD patients, who were asymptomatic with pyuria being seen in only about 60% of patients.^[20]^ Therefore, urine culture was essential and should be repeated. In this study, blood cultures were obtained at least 3 times in all the patients when admission. The most prevalent uropathogen identified was *E coli* (46.67%) followed by *K pneumoniae* (13.33%) and *E faecalis* (8.89%). Most gram-negative isolates were sensitive to imipenem, tigecycline, and piperacillin/tazobactam, while gram-positive isolates were sensitive to linezolid, teicoplanin, and vancomycin. However, we found that antibiotic resistance of urinary tract pathogens was particularly serious in patients with HBV-ACLF. Prevalence of MDR and XDR bacterial infections was 53.33% and 5.19%. The reason could be that our hospital was a higher-level referral hospital instead of a primary medicare one, so the patients usually comprised a high number of critically ill and some of them were given prophylactic antibiotics. Therefore, we should choose the most appropriate anti-infective therapy according to the results of the antibiotic susceptibility test and the general condition of the patients. In fact, at least 3 or more antibiotics were applied in approximately 50% of the patients with HBV-ACLF and the time of duration were sustained over 20 days in > 50% of the patients. Fernández et al^[21]^ reported that bacterial infections could promote a burst of systemic inflammation that precipitated the development of the syndrome of ACLF. In our investigation, the parameters of systemic inflammation (WBC count, serum C-reactive protein, ferritin, and interleukin-6) were also collected. Compared with the noninfection group, patients with infections showed higher levels of WBC (7.30 ± 3.92 vs 6.61 ± 3.24 × 10^9^/L; *P* = .029) and C-reactive protein (20.30 ± 18.81 vs 12.98 ± 11.16 mg/L; *P* = .001), and the other parameters had no significance (ferritin: 2680.50 ± 4161.74 vs 2242.63 ± 1609.55 μg/L; *P* = .354; interleukin-6: 53.06 ± 86.30 vs 33.63 ± 81.50 pg/mL; *P* = .386). Since the parameters of systemic inflammation other than WBC have not been detected in all the patients, the data were incomplete, which only seemed to be for reference and were not shown in our results.

Some research indicated that the incidence of UTI was higher in females of ESLD patients, and there was a positive association between primary biliary cirrhosis and UTI.^[20,22]^ But debate still existed as to whether women with primary biliary cirrhosis were at a higher risk of UTI.^[23]^ Therefore, logistic regression analysis was used for modeling the relationship between UTI and clinical characteristics, in order to investigate the risk factors of UTI in HBV-ACLF patients. Compared with the non-UTI group, patients with UTI showed higher levels of SCr, lower educational status, TBIL, DBIL, and albumin. This implied that UTI was determined by a diverse set of complex factors in patients with HBV-ACLF. Eventually, we found that lower educational status and hypoalbuminemia were associated with higher risk of UTI. In the study, 91.61% of the patients with UTI had low and middle educational status, which resulted in being performed as manual work including farmer and worker. This may be attributed to low economic status, poor living environment and habits, limited knowledge. Javaheri et al^[24]^ have reported that developing an education program was practical and useful in improving the preventive behaviors of patients from having UTI. Meanwhile, the majority of the patients with lower educational status usually have unsatisfactory knowledge regarding UTI. When they feel uncomfortable, they often choose to take their own antibiotics rather than to go to the hospital to consult a doctor. As a matter of fact, inappropriate antimicrobial use due to the lack of adequate knowledge about drugs can lead to inadequate therapy and contribute to further drug resistance.^[11]^ Hypoalbuminemia played another important role in the incidence of UTI in patients with HBV-ACLF. First, low albumin level is a predictor of morbidity/mortality regardless of the implicated disease. A decreased serum concentration of albumin can be caused by impaired liver synthesis, which is also available to evaluate the severity of liver and immune dysfunction.^[25]^ Hayes and Abraham^[26]^ have reported that a rapid and vigorous response mediated by the immune system was largely responsible for guarding against bacterial infections that bypass the natural defenses of the urinary tract. However, the capability was greatly weakened in state of immunological deficits. Second, under- or malnutrition has been proven to be associated with an increased risk of infection, and albumin could be used as a marker of adult malnutrition evaluation.^[27]^ Third, low albumin level can also alter the inflammatory response in a number of ways, including scavenging and modulation of cytokine production,^[28]^ and some studies found that hypoalbuminemia was a risk factor related to the onset, complications, length of hospitalization, and mortality caused by various infections.^[29]^ Systemic inflammation may increase bacterial translocation. The secondary release of norepinephrine at the intestinal mucosa impairs the local immune system function and induces bacterial translocation.^[21]^ In addition, translocation is also facilitated by the increased intestinal permeability that occurs with bowel edema and portal hypertension. When the bacteria outgrow the capacity of lymph nodes, they spill over into the bloodstream.^[30]^ The evidence shows that uropathogenic *E coli* was responsible for >80% of UTI and the pathogen was believed to originate from the intestines as these bacteria could be regular components of the microbiome of the gastrointestinal tract.^[26,31]^ Moreover, patients with lower albumin are usually complicated with ascites and less urinary output, which is more susceptible to UTI.

Unlike uncomplicated UTI, treatment of UTI in patients with HBV-ACLF depends on a series of factors (such as compromised immune system, severity of illness, and risk for multidrug resistance). Therefore, prognosis of UTI in patients with HBV-ACLF was heterogeneous. Some patients can achieve obvious improvement, but other may have delayed or incomplete response to therapy in underlying medical conditions. Patients who developed pyuria or were at increased risk for recurrence needed closer monitoring. Repeated urine tests and urine cultures were necessary as a result of most asymptomatic patients with UTI.

This study still has several potential limitations. First, although we collected clinical outcomes of all patients in UTI group and non-UTI group, because some patients were discharged automatically during treatment, the specific outcomes were not completely clear, which may have a certain impact on the true prognosis. Second, it was a single-center study and the number of patients with HBV-ACLF were limited. Finally, some data on systemic inflammation were incomplete, so the whole evaluation for bacterial infection in patients with HBV-ACLF was weakened. Hence, further studies with multiple centers and larger samples should be conducted in the future.

In conclusion, UTI is one of the common bacterial infections seen in patients with HBV-ACLF. gram-negative bacteria account for the majority of cultured bacteria, and MDR bacteria are common. Given increasing antimicrobial resistance, regular use of antimicrobials to prevent UTI may not be appealing. We recommend that patients with MDR who are severely ill should receive initial therapy with a carbapenem and this should be tailored once susceptibility is known. UTI is determined by a diverse set of complex factors, which lower educational status and hypoalbuminemia predict the more prevalence of UTI. Therefore, clinicians should rule out all the complicated factors to determine individualized treatment. The findings further the epidemiology profile of UTI across the spectrum of HBV-ACLF and will be beneficial in the management of patients with HBV-ACLF.

## Acknowledgments

The authors are indebted to Zheng-Ce Wan and Li Liu for assistance with the methodology.

## Author contributions

Qian Zhang conceived and designed the experiments, performed the experiments, analyzed the data, prepared figures and/or tables, authored or reviewed drafts of the article, and approved the final draft. Baoxian Shi performed the experiments, analyzed the data, prepared figures and/or tables, and approved the final draft. Liang Wu conceived and designed the experiments, authored or reviewed drafts of the article, and approved the final draft.
